# Microbiological quality of water sources in the West region of Cameroon: quantitative detection of total coliforms using Micro Biological Survey method

**DOI:** 10.1186/s12889-020-8443-0

**Published:** 2020-03-17

**Authors:** Rodrigue Mabvouna Biguioh, Sali Ben Béchir Adogaye, Patrick Martial Nkamedjie Pete, Martin Sanou Sobze, Jean Blaise Kemogne, Vittorio Colizzi

**Affiliations:** 1 Faculty of Medicine and surgery, University of Roma “Tor Vergata”, Rome, Italy; 2 Institute for Research, Socio-Economic Development and Communication (IRESCO), Yaoundé, Cameroon; 3 Faculty of Medicine and pharmaceutical Sciences, University of Dschang, Dschang, Cameroon; 4 Evangelic University of Cameroon, Mboua-Bandjoun, Cameroon

**Keywords:** Micro biological survey, Water microbiological analysis, MBS-HACCP & water easy test®, Water points, West Cameroon

## Abstract

**Background:**

Adequate supply of safe drinking-water remains a critical issue in most developing countries. The whole western region of Cameroon doesn’t have a sustainable continuous water supply system, which leads most people to use potentially contaminated water sources to meet their daily water needs. Previous, studies carried out in similar areas of Cameroon have highlighted the poor bacteriological quality of water sources used as drinking-water by the local populations.

**Methods:**

This study used the Micro Biological Survey method, a rapid colorimetric test for the quantitative detection of Coliforms in water samples. 22 water sources (12 improved and 10 unimproved) were identified; 1 water sample of 50 ml was collected in sterile plastic tubes, immediately kept in a refrigerator box and transported to the laboratory for analysis. 1 ml of each sample was inoculated in the Coliforms Micro Biological Survey (Coli MBS) vials initially rehydrated with 10 ml of sterile distilled water. The Coli MBS vials were closed, shaken for about 30 s for homogenization and then incubated at 37 °C. From the initial red color of the Coli MBS vials, changes in color of the reaction vials were monitored at three different time intervals (12 h, 19 h and 24 h), corresponding to three levels of contamination.

**Results:**

The average distance (8.7 m) of the latrines from the nearest water source was less than the minimal recommended distance (15 m) to ovoid external contamination. The pH of water samples ranged from 5.5 to 8.3 and the maximum temperature found (26 °C) was almost at level favorable to outbreaks of waterborne diseases such as cholera. The presence of Total Coliforms was detected in 90.91% of the samples. 40% of samples were positive 12 h after the analysis beginning. High level of contamination was observed in unimproved water sources, 50% after 12 h corresponding to Total Coliforms concentration of 10 < x < 103 CFU/ml and the other samples after 19 h (Total Coliforms concentration: 1 < x < 10 CFU/ml).

**Conclusion:**

This study revealed the poor microbiological quality of water used by local populations of our study sites. There is need to conduct further qualitative microbiology studies to isolate potential germs involved in outcome of diarrheal diseases.

## Background

Safe drinking-water is an essential resource for human life, a basic human right and one of the key components of effective health protection policy [1, 2]. It is known that drinking-water is one of the main transmission pathways for diarrheal diseases [2]. It is also established that improving the bacteriological quality of drinking-water significantly reduces the risk of waterborne diseases [3]. Thus, every effort should be made to achieve a satisfactory drinking-water supply to all in terms of adequacy, safety and accessibility [2].

Most developing countries face high population growth which poses a considerable challenge for local authorities who are not able to meet basic needs of populations whose most crucial problem is sustainable access to save drinking-water [2, 3]. Ensuring availability and sustainable management of water sources and sanitation for all, remains one of the most important Sustainable Development Goals governments need to achieve [4]. According to the World Health Organization (WHO), safe drinking-water does not represent any significant risk to health over a lifetime of consumption, including different sensitivities that may occur between life stages [2]. Therefore, microbial, chemical and other acceptability aspects of drinking-water should feet with WHO guidelines for drinking-water quality [2].

Supply of safe drinking-water for human is a critical problem in African countries [5–7], more importantly in remote areas due to the hyper centralization of public management services [8, 9]. As in other parts of the country, but less in the Central or Littoral regions, the West Cameroon does not have a continuous water supply system, leading to majority of people to use surface, well, borehole and river water as alternative source of drinking-water and for other water needs [8, 10]. Studies carried out in similar areas of Cameroon have highlighted the poor bacteriological quality of these water sources [11–14].

Waterborne diseases are the second leading cause of death and infant morbidity after malaria in Bafoussam and in other main cities of the West Cameroon [15], indicating the non-achievement of bacteriological standards of drinking-water standards for human consumption [16]. Morbidity and mortality rates of diarrheal diseases are more prevalent among children under 5 [15]. Among the top 10 diseases in children under 5 including malaria, infection of the lower respiratory tract, upper respiratory tract infection, meningitis, typhoid fever, bloody diarrhea, diarrhea (non-bloody diarrhea), dysentery, parasitic worm infection and gastritis, 4 of them are related to the consumption of unsafe water and/or food [15, 16]. The germs responsible for these diseases are generally transmitted by feco-oral route and represent a major concern in public health risk [16]. Microbiological contamination of water occurs in a context of poor waste management including faeces [17]. Contaminated water with bacteria should not be intended for human consumption. Coliforms can be used as indicator to monitor the microbiological quality of drinking-water [18, 19] and their rapid detection is therefore crucial and should be easy to perform in order to evaluate water quality especially in resource limited countries, such as Cameroon. Preventive public health approaches for safe drinking-water must include rapid assessment of microbiological quality of water to guide monitoring of water quality and treatment. In line with this point of view, this study aimed to assess the potability of water points using Micro Biological Survey Hazard Analysis Critical Control Point (MBS-HACCP) & water Easy test® [20] in the West Cameroon.

## Methods

### Study area and sampling

**Fig. 1:**
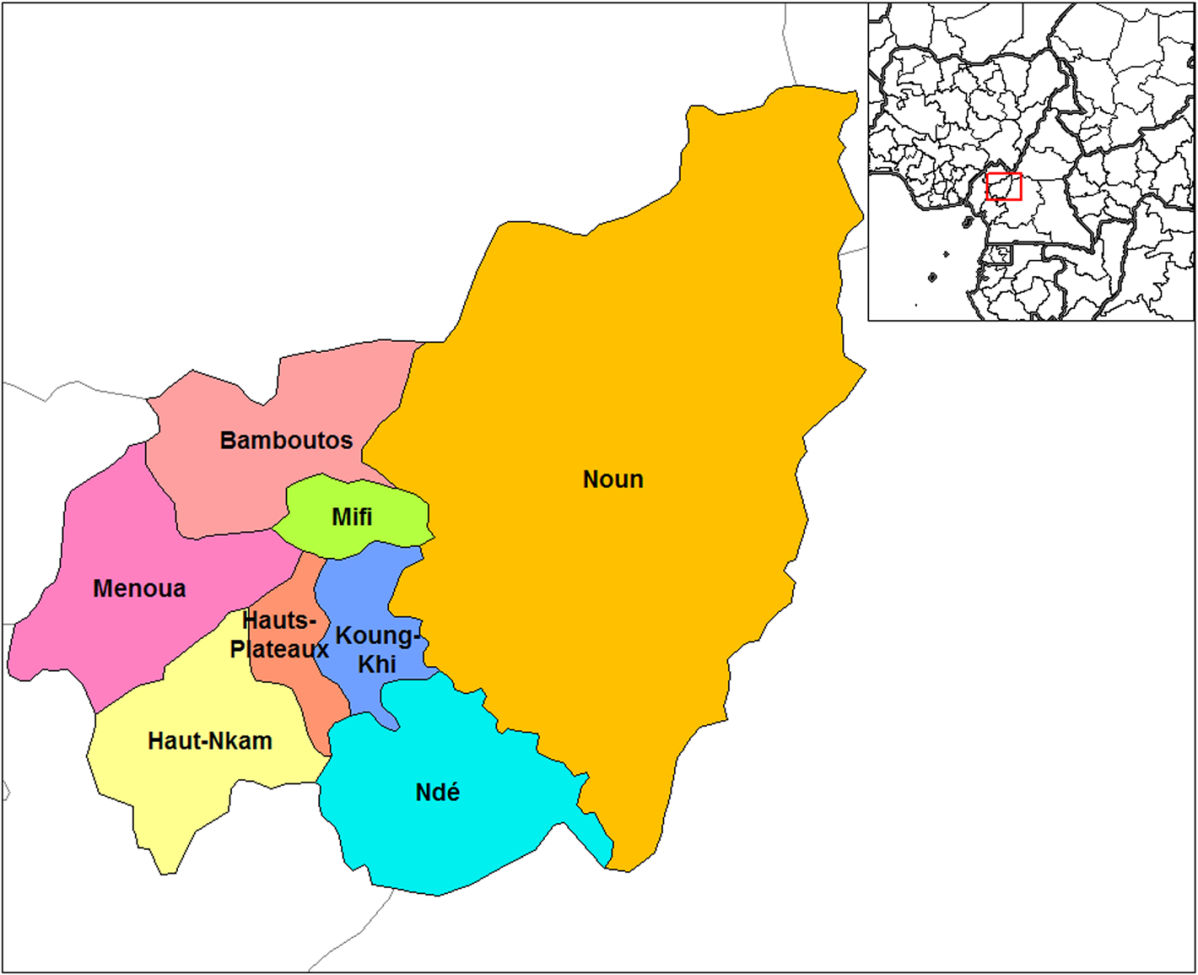
Map of the Western Region of Cameroon. Source: Wikimedia Commons. https://commons.wikimedia.org/wiki/File:West_Cameroon_divisions.png

The study was performed in West region of Cameroon (Fig. 1), an area of 14,000 km^2^ located in the central-western part of the country with a total population estimated at 1,921,590 inhabitants [21]. Even if the region is the smallest of the country in terms of area, the West region has the highest population density (140/km^2^). The climate is mainly cold with an average temperature varying between 15 and 22 °C, sometimes reaches 30 °C during the dry season and rainfall is moderate.

A water source was considered as improved if the nature of its construction satisfactorily protects the water from any external contamination, especially faeces. 12 improved and 10 unimproved water sources were identified in the study area, especially in the localities of Bafoussam 1^er^, Bafoussam 2^e^, Foumbot, Galim and Kouoptamo (Table 1). At each site, 1 water sample of 50 ml was collected in sterile plastic tubes and immediately kept in a refrigerator box and transported to the laboratory for microbiological analysis.

**Table 1:** Sampling location and type of water sources

Sampling location	Water sources	Sample collection date
**Bafoussam 1er**	Unimproved water spring	23/09/2018
	Unprotected well	25/09/2018
	Public water drilling	28/09/2018
**Bafoussam 2e**	Improved water spring	24/09/2018
	Protected well	28/09/2018
**Foumbot**	Improved water spring	23/09/2018
	River	24/09/2018
	Improved water spring	24/09/2018
	Unprotected well	24/09/2018
	Protected well	24/09/2018
	Protected well	24/09/2018
	Unprotected well	24/09/2018
**Galim**	Protected well	23/09/2018
**Kouoptamo**	Unprotected well	23/09/2018
	Unprotected well	25/09/2018
	Unprotected well	25/09/2018
	Protected well	25/09/2018
	Improved water spring	28/09/2018
	Unprotected well	28/09/2018
	Improved water spring	28/09/2018
	Unprotected well	28/09/2018
	Protected well	28/09/2018

Water sources in this study were located on areas of public accessibility and are used for free by local population. The 12 improved sources were constructed by volunteers, but they don’t have authority over these water points in terms of management and control. So, for the collection of water samples, no authorization was requested.

### MBS-HACCP, water easy test and MBS method

The MBS-HACCP & water Easy test is a rapid colorimetric test for the quantitative detection of coliforms in water samples [20]. The method is based on observation of color change of the suspension formed in the analysis vial following inoculation of the test sample. The color change occurs when the water sample added in the vial contains Coliform bacteria, the greater the amount of microorganisms, the more rapid the color change and thus, a positive result (contamination). The concentration of bacteria is expressed in Colony Forming Units (CFU/ml) for the analysis water samples. The MBS method is based on the detection of bacterial metabolism and not on the replication of microorganisms, but the results (in terms of number) correspond to individual bacterial cells.

### Water analysis: MBS operating procedures for quantitative detection of Total coliforms

We followed the MBS standard protocol for quantitative detection of Total Coliforms [20]. Before starting with the analysis, the MBS vials were rehydrated with 10 ml of sterile distilled water and shaken to dissolve the reagent. 1 ml of each sample was collected from plastic tubes using a sterile Pasteur pipette and inoculated in the Coli MSB vial. The vials were then closed and shaken for about 30 s for homogenization. Each analysis was done twice, the vials were incubated at 37 °C. The color changes of the Coli reaction vials were monitored using the chromatic scale provided with the tests at three different time intervals (12 h, 19 h and 24 h), corresponding to three levels of contamination. The initial color of the Coli vials is red. A color change from red to yellow after 12 h indicates a very high contamination (Total Coliforms concentration >  10^3^ CFU/ml), a color change at 19 h indicates a high contamination (Total Coliforms concentration 10 < x < 10^3^ CFU/ml) and a color change at 24 h corresponds to Total Coliforms concentration of 1 < x < 10 CFU/ml [20].

## Results

### Description of water points

Water samples were collected in 22 sites. Improved water sources accounted for 54.55% (*n* = 12) while 45.5% (*n* = 10) were unimproved. The average distance of the latrines from the nearest water source was 8.7 m. The closest and the farthest water sources were at 1 and 100 m respectively from the nearest latrine. The pH of the water samples collected ranged from 5.5 to 8.3 and the temperature varied from 22 to 26 °C. Most of water sources were clear (90.91%) and the turbidity was noticed in both cases (Table 2).

**Table 2:** Summary of the water sources parameters

		Types of water sources		
		Improved	Unimproved	Overall
Average pH		6.7 ± 0.3	6.2 ± 0.7	6.5 ± 0.6
Average temperature (°C)		23.2 ± 0.6	23.5 ± 1	23.3 ± 0.8
Average distance to nearest latrine (m)		5 ± 2.7	13.3 ± 30	8.7 ± 20.5
Color of water	*Clear (%)*	12 (100)	8 (80)	20 (90.91)
	*Yellowish (%)*	0	2 (20)	2 (9.09)
Turbidity	*No (%)*	12 (100)	8 (80)	20 (90.91)
	*Yes (%)*	0	2 (20)	2 (9.09)

### Total coliforms samples contamination

**Fig. 2:**
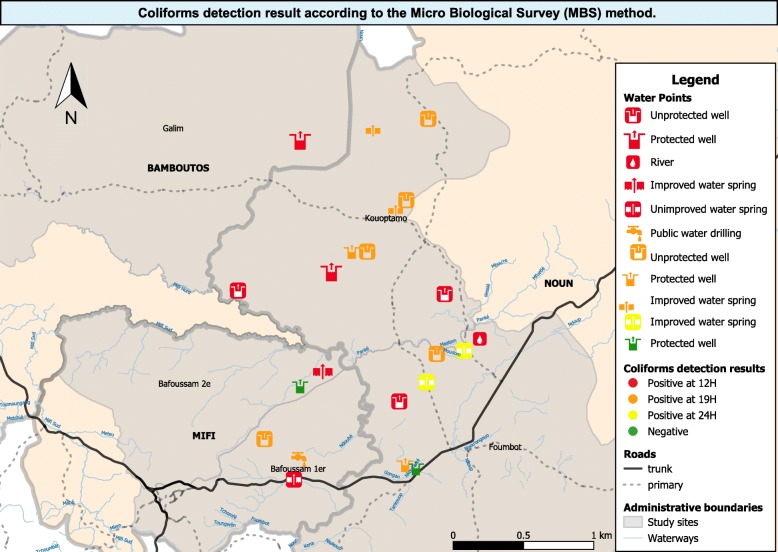
Coliforms detection result according to the Microbiological Survey (MBS) method. Source: Authors. Generated by QGIS version 3.4.11-Madeira (GNU GENERAL PUBLIC LICENSE Version 2, June 1991)

Of the 22 water samples analyzed, 20 (90.91%) contained Coliforms (Fig. 2). Both improved and unimproved water sources were evenly contaminated with Coliforms. All water samples with yellow coloration were positive as well as 90% of clear water samples. This result was similar for the turbidity.

### Total coliforms levels of contamination in water samples analyzed

Of the 20 samples positive for Coliforms, 40% (*n* = 8) were highly positive, indicating a very high level of coliforms contamination (Total Coliforms concentration >  10^3^ CFU/ml). Of all positive samples, high level of contamination was observed in unimproved water sources, 50% at 12 h corresponding to Total Coliforms concentration of 10 < x < 10^3^ CFU/ml and the other samples after 19 h (Total Coliforms concentration of 1 < x < 10 CFU/ml). Of all the water sources, only two samples (collected in improved water sources) were positives at 24 h (Total Coliforms concentration of 1 < x < 10 CFU/ml) (Table 3).

**Table 3:** Summary of total Coliforms contamination results according to monitoring time points

	Types of water sources					
	Improved		Unimproved		Total	
Monitoring time points and Coliforms concentration	n	%	n	%	N	%
12 h *(> 10*^*3*^*CFU/ml)*	3	30	5	50	8	40
19 h *(10 < x < 10*^*3*^*CFU/ml)*	5	50	5	50	10	50
24 h *(1 < x < 10 CFU/ml)*	2	20	0	0	2	10
**Total positive samples**	**10**	**100**	**10**	**100**	**20**	**100**

## Discussion

Safe drinking water is essential for life. This study aimed at analyzing the presence of bacteria, through quantitative detection of Total Coliforms in both improved and unimproved water sources in the West region of Cameroon. Inhabitants of the study area are in majority poor with limited capacities including financial to afford pipe borne water [8, 15], they turn to health threatening and potentially highly polluted water sources which could explain why diarrheal diseases mostly occur in populations with limited financial means. This has been described by a study conducted in South Africa, which highlighted that cholera outbreak does not on results from inadequate sanitations, but also due to poverty [22].

According to the WHO guidelines for drinking-water quality, the microbial safety of drinking-water includes the prevention of the drinking-water contamination by the microorganisms or the reduction of contamination to levels not injurious to human health [2]. While ingestion of microorganisms from contaminated water and food is the main cause of diarrheal diseases [23], lack of safe drinking-water is one of the leading causes of death especially in children under 5 [15, 16]. Total Coliforms are used as indicators of faecal pollution, the effectiveness of water filtration or disinfection, the integrity and cleanliness of water distribution systems [24, 25]. The WHO guideline for drinking-water quality recommends the absence of Total Coliforms in drinking-water [2]. This study highlights poor quality of the water due to the presence of Total Coliforms in high concentration. In fact, of the 22 water samples collected, 20 contained coliforms which could be associated with high risks of diarrheal diseases outbreaks, such as cholera as suggested by previous studies that stipulate fecal coliform-contaminated water may contain *Vibrio Cholerae* [26, 27]. Both types of water sources (improved and unimproved) were contaminated indicating a possible human or animal faecal pollution of these water points.

The minimal recommended distance between latrine and water source to avoid external contamination ranged between 15 and 50 m [2, 28]. The average distance found in this study did not feet with this recommendation. Even though improved water sources were more closely to latrines, unimproved water points which are open water sources showed greater concentration of Coliforms. This may be explained by the nature of the improved water sources construction satisfactorily protects the water from any external contamination [29]. This is consistent with previous studies which suggest that unprotected water sources have high probability of being contaminated by fecal material carried out by run-off water mainly during rainy season [22, 30]. Globally, the results of this study reveal poor quality of water sources used by the population. Our results are consistent with previous studies carried out in the West Cameroon and similar areas which indicated an alarming lack of safe drinking-water [8, 11–14]. Water samples with a small amount of germs turned pathogenic after 24 h of incubation. This maximum incubation time does not deviate from the WHO recommendations whose procedures include membrane, filtration followed by incubation of the membranes on selective media at 35–37 °C and counting of colonies after 24 h [2].

There is no pH guide value but an optimum between 6.5 and 9.5. The average water pH found in this study was 6.5 which correspond to the lower value recommended by WHO [2]. The temperature of the samples varied between 22 and 26 °C. This indicator needs to be monitored to support preventive public health to control measures, as it has been established that outbreaks of waterborne diseases such as cholera are consistent with a rise in temperature during the dry season and the peaks are reached in the rainy season [30]. Monitoring temperature and pH variations during the seasons could help in the planning and implementation of outbreaks prevention measures.

Microbial and other water constituents can affect the appearance of the water [2]. Most samples were clear (90.91%), suggesting their good quality and acceptability based of this criterion, but the Total Coliforms analysis have shown another case of figure indicating that changes in the normal appearance of water is not a sufficient signal of the water quality.

## Conclusions

This study revealed the poor microbiological quality of the water sources used by inhabitants of West Cameroon. These poor water sources could be at the origin of waterborne disease outbreaks. Even though qualitative analysis was not performed, the MBS method detected the presence of Coliforms in almost all the water samples collected. It is also important to emphasis that the quantity of Coliforms found in the samples could indicate the presence of disease-causing bacteria such as *Vibrio Cholerae*. The average distance (8.7 m) between the water point and the nearest latrine doesn’t meet up with WHO recommendations (15–50 m to the nearest latrine), showing groundwater high risk of contamination by faeces infiltration. There is need for the local public health services and rural council to establish local water management committees to help in monitoring and ensure water sources do not represent a risk of waterborne disease outbreak. In addition, local populations need to be trained on simple and cost-effective of water treatment techniques. Additional qualitative microbiology studies need to be conducted to isolate germs involved in diarrheal diseases.

## Data Availability

The datasets used and/or analyzed during the current study are available from the corresponding author on reasonable request.

## Abbreviations

HACCP: Hazard Analysis Critical Control Point
MBS: Micro Biological Survey
WHO: World Health Organization
